# Choice of provider for out-patient treatment is not working

**DOI:** 10.1192/bjb.2017.25

**Published:** 2018-04

**Authors:** David Veale

**Affiliations:** 1 South London and Maudsley National Health Service Trust, UK

## Abstract

In 2012, the coalition government introduced the legal right for National Health Service (NHS) patients in England to choose their mental healthcare provider for out-patient treatment. This was a significant step towards parity of care between mental and physical ill health, and NHS Improvement and NHS England have provided sensible guidance on its implementation. However, several factors prevent the system from working as it was intended: lack of awareness of the principle of patient choice for out-patient treatment; complacency from some trusts and commissioners; mental health trusts operating outside the e-referrals system; misuse of care pathways; lack of direct access by many general practitioners for out-of-area referrals; and delays in authorisation for funding. I describe how I have come to this conclusion and suggest some solutions.

**Declaration of interest:**

The author is employed by a trust that potentially benefits from ‘patient choice’.

The Royal College of Psychiatrists has campaigned for parity of care between mental and physical ill health and has secured some notable success in raising awareness and pushing mental health up the political agenda. One issue in parity of care, which remains problematic, is the right of patients to choose their out-patient provider in defined circumstances. Thus, if you visit your general practitioner (GP) with a cancer, you can choose your treatment provider, using a variety of criteria such as reputation, outcomes, waiting times and location. Paradoxically, choice is not often a priority in physical healthcare.^1^ When surveyed, people most value proximity, a short waiting list and ease of transport to healthcare. This is perhaps because the quality of physical healthcare is more similar across providers compared with, say, the delivery of a protocol in cognitive–behavioural therapy (CBT) for a particular disorder.

## Background

In 2012, the Rt Hon. Norman Lamb and the coalition government introduced the legal right for National Health Service (NHS) patients in England to choose their mental healthcare provider for out-patient treatment.^2^ This was a significant step towards parity of care and allowed patients suffering mental disorder to choose their *out-patient* provider, in the same way as they could if suffering from a physical condition, without the need for discussion at a funding committee. Older readers will remember that choice for mental health out-patient treatment used to be available on the NHS up until the early 1990s.

The way the change in law is interpreted is outlined in the document *Choice in Mental Health*, published by NHS Improvement, who are now responsible for the regulation of patient choice.^3^ NHS England have also published some helpful documents and guides on patient choice, from which some of this article is drawn.^4^^–^^6^

The guidance by NHS Improvement and NHS England is clear. Nevertheless, introduction of the change is being hampered by several factors: lack of awareness on the part of both professionals and the community; poor commitment by trusts and commissioners; and various loopholes that I will go on to describe. As it remains cheaper for a patient to be treated by a block contract with a local provider, some of the apparent lack of compliance may flow from an effort to contain costs. To follow the guidance for patient choice, out-patient treatment needs to follow a typical package of care at a service that has a standard NHS contract. This can be at two levels of stepped care.

### Primary care – Improving Access to Psychological Therapies (IAPT)

This consists of a psychological therapy with no consultant involvement. Thus, legally the GP and patient may decide they wish to go to a different provider even if a clinical commissioning group (CCG) has a block contract with a local IAPT service.

### Secondary care – consultant-led service

Where a patient with moderate to severe symptoms requires a more experienced therapist and medication advice in a consultant-led service, this can also be accessed under patient choice by a GP. The treatment must be *clinically appropriate*, without the need for local community mental health team (CMHT) involvement or integrated social care. Here, the service acts as the secondary care provider. Again, under patient choice, the existence of a care pathway with a block contract for a local CMHT or psychological therapy service is irrelevant if the GP and patient decide they wish to go to a different provider. Clarity over the right of patients to choose may be particularly important where there is a need for assessment or treatment in one of the many ‘Cinderella’ services in psychiatry, such as anxiety disorders and trauma, attention-deficit hyperactivity disorder (ADHD), chronic fatigue or medically unexplained symptoms, where local services may not be well developed. It might involve out-patient child and adolescent services if other local services are not required. However, there is no obligation on the provider of a CMHT to travel to the patient; in effect, choice depends on the patient traveling to an out-patient provider.^5^

In brief, NHS England^5^ state that a patient is *not* entitled to choose where to be referred in the following cases.

1 The patient's needs cannot be addressed by a typical package of care that lies outside National Institute for Health and Care Excellence (NICE) guidance or is not routinely commissioned on an ongoing basis through a NHS standard contract. An example for which choice of provider does not exist might be repetitive transcranial magnetic stimulation for depression, because it is not routinely commissioned. An example of where choice would exist is CBT for post-traumatic stress disorder (PTSD), which would be commissioned to be treated at a local IAPT or secondary care service.
2 The patient's needs mean that an outside referral is *not* clinically appropriate. For example, urgent care for a patient with significant risk factors (such as self-neglect or a risk of suicide), which are best served by local CMHT involvement. Patients cannot choose their level of care (such as residential or in-patient over out-patient care) unless it is clinically appropriate (for example, intensive CBT on a residential unit for severe obsessive–compulsive disorder). Nor are patients entitled to choose a non-local provider if treatment is likely to need integrated care with social services or a CMHT because of the level of risk. These are examples of where it is not clinically appropriate for a GP to refer that patient to an out-patient service.

If patients need a local CMHT, then the GP cannot refer to a specialist service. If appropriate, the CMHT will make an individual funding request to a CCG for assessment or treatment at a national specialist service as part of tertiary care.

The principles developed by NHS Improvement and the Patient Choice department of NHS England5 are sensible, but parity of care is not working in practice. I highlight some of the key problems and possible solutions below.

## Lack of awareness of patient choice on the part of patients and GPs

Patients and GPs remain broadly unaware of patient choice. It seems very strongly embedded in the culture that mental healthcare is differently regarded from physical care and that only in exceptional cases can patients be referred out of area. I have read refusals for funding from commissioners in mental health, which cite unapologetic expectation that routine patients be treated in a locally commissioned service. I have lost count of the patients who report that their GP swiftly dismissed any suggestion that an out-of-area referral is possible other than in exceptional circumstances (i.e. a tertiary care option) or where ‘local resources have been exhausted’. On the NHS Choices website, psychiatric hospitals are commonly listed as providing a service for their local population. There is no listing of, say, a clinic for anxiety disorders, ADHD or eating disorders that will accept referrals from GPs all over England.

Linked to this problem, the NHS Choices website specifies no waiting times, or details of outcomes, awards or patient feedback for any individual clinic. Neither do hospital or IAPT websites make clear when a service can receive a direct referral from a GP from around the country or when it requires a CMHT to make the referral. Where patient-reported outcome measures (PROMs) are available, presentation is very patchy (and tends to refer to an IAPT service in one borough, rather than a service like our own at the Centre for Anxiety Disorders and Trauma which accepts a selection of patients across different IAPT services). There appears to be little desire by trusts to develop special interests, and so trusts that do provide a ‘specialist’ service appear to regard them still as only available in complex cases or for tertiary care, for which patient choice does not apply. This is not the case. Services such as our own not only have funding streams for treating patients with severe cases in tertiary care but also treat patients within primary care (IAPT) and secondary care as described above. NHS Improvement published further guidance earlier this year in user-friendly language. The leaflets^5^ were sent directly to all CCGs.^2^ Although Mind and other mental health charities also publicised the initiative, the message is still not getting through to GPs or patients who would like the option of choice for out-patient treatment where it is clinically appropriate.

It would undoubtedly help if NHS Digital and providers were to publicise on their websites those services that are directly available to GPs, accompanied by clear data. At present, NHS Choices tends to describe the hospital but not individual clinics, although I acknowledge they are dependent on the information provided. NHS Choices does, however, provide a list of private therapists, which seems odd on an NHS website. ‘Specialist’ services on hospital websites appear to be solely relevant for tertiary care rather than open for GP referrals. The NHS Choices and individual hospital websites need to encourage the reporting of standard PROMs and other meaningful information such as waiting lists for individual clinics. Patients find it difficult to make choices unassisted and currently seem to rely mainly on reputation and charity helplines. This is not good enough.

## Mental health foundation trusts are not part of ‘e-referral’ systems on GPs’ computers

Only a small number of mental health providers have joined e-referral (or the old ‘Choose and Book’ system) in the same way as physical care providers do. This prevents GPs from using the system as intended, while feeding the misperception that they are obliged to use the local service. Thus, if you have a physical health problem, your GP can book you into a clinic of your choice on his/her computer. Waiting times are generally known for competing hospitals providing physical healthcare. Mental health trusts appear to prioritise their CMHT services over implementation of e-referrals and other mechanisms to assist with the guidance.

## Misuse of care pathways

A ‘care pathway’ is a way of describing *how* a patient is assessed and treated for a specific problem. Many hospital trusts and CCGs have care pathways which are helpful. They are generally based on NICE guidance, although there are often areas in which there is not sufficient evidence and a pathway is based on other evidence and expert opinion. Care pathways are sensibly used for long-term physical conditions such as diabetes, which require local joined-up solutions. However, care pathways in mental healthcare are often misused; they may state *the location* of a local service without highlighting the need to discuss patient choice when there are no significant risk factors or any need for local social services or a CMHT (as required by NHS Improvement). Other misuses of care pathways include referrals for a patient with a certain condition (e.g. chronic fatigue) being directed to a specific care provider that has a block contract, with no discussion about choice.

My view is that NHS Improvement needs to ensure that, when a patient does not have significant risk factors (e.g. suicide, neglect) or any need for integrated social services, a local care pathway includes a discussion of choice and relevant documents are easily available on a local website (rather than, for example, obtained under the Freedom of Information Act). Here, NICE could highlight the role of choice in out-patient treatment for problems that do not require local CMHT or social services.

## Lack of direct access by GPs to IAPT providers out of their area

Increasingly, CCGs prevent their GPs from directly referring to a non-local IAPT provider. GPs may refer to a local IAPT service, but anything else is directed to a local triage assessment or to a funding panel. In other words, not all providers are treated equally, and delays are created for patients referred to triage or a funding panel. Triage can be helpful when a patient is likely to need a local CMHT (for example, if a patient has significant risk factors or is likely to need mental healthcare integrated with social services). An IAPT patient by definition does not have significant risk, so this is an example of inherent bias (or lack of parity). Triage is not required for referral to a local IAPT level of service, but it may be required to determine whether the patient needs to be referred to a CMHT. Indeed, people are entitled to self-refer without even needing to involve a GP, yet if they have the temerity to go out of area, they have to be triaged to discuss the benefits of the local IAPT service, thus delaying treatment.

At present, NHS Improvement allows CCGs to set up care pathways as they see fit. They believe that a triage assessment can be used to determine whether the referral is clinically appropriate, so long as choice is discussed. However, local triage services do not collect the relevant PROMs or waiting times, or keep a current list of specialised clinics of other IAPT providers around the country. Sample documents have shown that it is not normally part of the policy to discuss choice of provider (and certainly choice of IAPT provider). Many CCGs just ban their GPs from referring out of area, and such requests have to go to a funding panel where the GP is required to request special circumstances, which is inconsistent with patient choice. In practice, patients conduct their own research and discuss it with their GP or hope that the GP has specialist knowledge and can advise them. IAPT services are perfectly capable of saying when treatment with them is not appropriate, without local CMHT involvement. Patients do not need another triage assessment – otherwise they would not be allowed to refer themselves directly to a local IAPT provider.

In summary, IAPT represents the lowest level of stepped care. Patients are often entitled to self-refer. The principle of a triage service for IAPT patients who wish to be referred to an out-of-area provider is therefore nonsense. In my view, NHS Improvement needs to ensure that IAPT patients can be assessed at the provider of their choice. Referrals to non-local providers need to state clearly which service is sought (for example, IAPT rather than secondary care).

## Lack of direct access by the GP to a secondary care provider

By secondary care provider we mean a consultant-led service which deals with more complex problems than IAPT does or where medication advice may be required. Again, GPs are often prevented from directly referring outside the area or are restricted in doing so until after triage by their local provider. Local triage services are appropriate when local CMHT or social services involvement is likely to be required. Again, NHS Improvement does not define how referrals should be managed *so long as choice is discussed* at a triage service. As we have seen above in discussing IAPT services, this is naïve: triage services do not have regular access to information about other services, relevant PROMs and care pathways. In practice, despite the guidelines, triage services do not discuss the importance of patient choice in their policy documents, and not all providers are treated equally. Alternatively, such referrals are just blocked at funding panels, and the patients and GPs are unaware of their rights.

In my view, NHS Improvement needs to ensure that all triage services include a full discussion of choice in their policy documents or care pathways, and that they can provide details of other relevant services. In addition, such cases should not be discussed at funding panels. Referrals to providers need to state clearly that they are seeking a secondary care service rather than IAPT, and that they require assessment and treatment by a consultant's team.

## Delays in authorisation of funding for assessment

When a patient is finally referred, there must be pre-authorisation for funding by the CCG. Referrals for mental disorders out of area are still widely treated as something unusual, and the funding panels question why the patient cannot be treated locally or use a care pathway that states where a patient must be referred (because that is where there is a block contract). There are many examples at my own service of patients referred by their GP who wait several months, even a year, for applications to be ignored, and then find that funding is never authorised. About 50 patients who have been referred by their GPs to my department at the Centre for Anxiety Disorders and Trauma are waiting for authorisation for funding from CCGs, either for an assessment or, when they have been assessed, for treatment. Of the 50 I recently audited, seven have been waiting more than a year, 13 for more than 36 weeks, 25 for more than 24 weeks, 38 for more than 12 weeks, and the rest for more than 4 weeks. The picture has been similar in previous years. We recently reviewed our list and found that some patients had given up: some accept local treatment; some seek private care they can't afford. The people who can choose the location of their treatment are either lucky (depending on their postcode), or extremely persistent and articulate, or helped by an advocate. Once a patient has been assessed by a provider and treatment is recommended, there may then be delays obtaining funding for treatment. This is wrong, because the guidelines direct that in such cases, the CCG concerned cannot then require the patient to switch to its own provider for treatment. Although the NHS Improvement guidance on this does appear clear, the message is still not getting through to CCGs. My own trust has hundreds of outstanding requests for authorisation of GP referrals for assessment or treatment (not just for our department). Much time is wasted by the trust and by patient advocates in chasing CCGs, and patients experience delays in treatment, resulting in avoidable distress to patients and their families. If these same patients were suffering a physical health problem and required out-patient treatment, no authorisation for funding would have to be made: an invoice for activity would just be presented at the end of a treatment episode. Funding should only be refused if the treatment is not clinically appropriate (and not, for example, because the applicant should be treated in the local care pathway), and any such process should not unduly delay assessment or treatment. GPs and patients can complain directly to the regulator at NHS Improvement (https://improvement.nhs.uk/contact-us/) if they feel they are being denied their rights.

## Conclusions

Some patients want to be able to choose their treatment provider for out-patient treatment. This will mainly affect more ‘specialist’ services such as anxiety disorders, chronic fatigue and ADHD, where a local service may be less well developed. They have a legal framework and guidelines from the regulator to support them. However, more than 4 years after the system was introduced, it is not working effectively to deliver the care intended.

## About the author

**David Veale** is a Consultant Psychiatrist at the Centre for Anxiety Disorders and Trauma, South London and Maudsley NHS Foundation Trust, UK, and Visiting Professor in Cognitive Behavioural Psychotherapies at the Institute of Psychiatry, Psychology and Neuroscience, King's College London, UK.
